# Prevalence, antimicrobial susceptibility and virulotyping of *Listeria* species and *Listeria monocytogenes* isolated from open-air fish markets

**DOI:** 10.1186/s12866-015-0476-7

**Published:** 2015-07-25

**Authors:** Hossein Jamali, Mohammadjavad Paydar, Salmah Ismail, Chung Yeng Looi, Won Fen Wong, Behrad Radmehr, Atefeh Abedini

**Affiliations:** Biohealth Science Program, Institute of Biological Science, Faculty of Science, University of Malaya, 50603 Kuala Lumpur, Malaysia; Department of Pharmacology, Faculty of Medicine, University of Malaya, 50603 Kuala Lumpur, Malaysia; Department of Medical Microbiology, Faculty of Medicine, University of Malaya, 50603 Kuala Lumpur, Malaysia; Department of Food Hygiene and Quality Control, Islamic Azad University-Karaj Branch, Karaj, 31485-313 Iran; Centre de Recherche en Reproduction Animale, Université de Montréal, C.P. 5000 St-Hyacinthe, QC Canada

**Keywords:** *Listeria*, Seafood, Virulence genes, Serotyping, Antibiotic resistance, Resistance gene

## Abstract

**Background:**

The aim of this study was to investigate the prevalence and characterization of *Listeria* species and *Listeria monocytogenes* isolated from raw fish and open-air fish market environments. Eight hundred and sixty two samples including raw fish and fish market environments (samples from workers’ hands, workers’ knives, containers and work surface) were collected from the open-air fish markets in the Northern region of Iran.

**Results:**

*Listeria* spp. was isolated from 104/488 (21.3 %) raw fish and 29/374 (7.8 %) of samples from open-air fish market environment. The isolates of *Listeria* spp. included *L. innocua* (35.3 %), *L. monocytogenes* (32.3 %), *L. seeligeri* (18 %), and *L. ivanovii* (14.3 %). Of the 43 *L. monocytogenes* isolates, 31 (72.1 %), 10 (23.3 %) and 2 (4.7 %) belonged to serovars 1/2a, 4b, and 1/2b, respectively. The *inlA, inlB, inlC, inlJ, actA, hlyA, iap, plcA,* and *prfA* virulence-associated genes were detected in almost all of the *L. monocytogenes* isolates. The *Listeria* spp. isolates showed high resistance against tetracycline (23.3 %), penicillin G, and cephalothin (each 16.5 %). Besides, we observed significant resistance level to tetracycline (27.9 %), ampicillin (20.9 %), cephalothin, penicillin G, and streptomycin (each 16.3 %) in the *L. monocytogenes* isolates. All of the isolates were susceptible to cefotaxime, gentamicin, kanamycin, and pefloxacin. We found that *tetM* (25.6 %), *tetA* (23.3 %)*, ampC* (14 %)*,* and *penA* (11.6 %) were the most prevalent antibiotic resistance genes in the *L. monocytogenes* isolates.

**Conclusions:**

Recovery of potentially pathogenic *L. monocytogenes* from raw fish and environment of open-air fish market samples in this study is a convincing evidence for the zoonotic potential of listeriosis.

## Background

The genus *Listeria* includes facultatively anaerobic Gram-positive bacteria. *L. ivanovii* and *L. monocytogenes* from this genus are known as pathogenic species and have shown the ability to cause severe diseases in animals and both humans and animals, respectively. Listeriosis is a foodborne infection with high mortality rates which is caused by *L. monocytogenes* in humans. The pathogen is ubiquitous and has been isolated from animals, different types of foods, and environments worldwide [1–3].

There is a high possibility of *L. monocytogenes* contamination in the captured fish from contaminated waters and environments. The contamination could also happen during transportation and in the environment of fish markets. Although *L. monocytogenes* has been isolated from seafood, fish and fishery products, no major listeriosis outbreaks with these products has been reported so far [4]. However, contaminated fish and fish products are considered as the most frequent causes of a number of sporadic listeriosis cases [5].

*L. monocytogenes* can be divided into 13 serovars which serovars 1/2a, 1/2b and 4b have been mainly reported from foods and human cases of listeriosis [6–8]. The 1/2a is a predominant serovar isolated from food samples [9]. However, serovar 4b is known to be the cause of the largest number of human listeriosis outbreaks [10, 11].

Different *Listeria* determinants, which are well known as important factors in pathogenicity of *L. monocytogenes*, include actin (encoded by *actA* gene), internalin (encoded by *inlA, inlB, inlC,* and *inlJ* genes), invasion associated protein (encoded by *iap* gene), listeriolysin O (encoded by *hlyA* gene), phosphatidylinositol phospholipase C (encoded by *plcA* gene), and virulence regulator (encoded by *prfA* gene) [12–15].

Detection of multi-drug resistant pathogenic bacteria in foods is considered as a public health risk worldwide. Excessive application of antibiotics in veterinary medicine may lead to distribution of antibiotic-resistant pathogens in the environment [16]*.* Recently, multi-drug resistant isolates of *L. monocytogenes* have been detected in animals, different types of foods and human cases of listeriosis [17–20]. Since multi-drug resistant isolates could transmit to humans via contaminated food, proper measures should be taken to prevent their environmental spread. Monitoring of the multi-drug resistant pathogens isolated from food and environment could be useful to identify the patterns of resistance to antibiotics [21].

Mazandaran province, which is located in the North of Iran and near Caspian Sea, fish and fish products are consumed more than other places in Iran. Despite the high consumption of fish and fish products in this area, there is no study on the prevalence and characterization of *Listeria* spp. and *L. monocytogenes* from fish and environment of fish markets. Hence, this study was done to investigate the prevalence, serotyping, virulotyping and the resistance patterns of *Listeria* spp. and *L. monocytogenes* isolates detected from fish and environment of fish markets in Mazandaran province and the Northern region of Iran.

## Methods

### Sampling

Between March 2012 and Jun 2014, 488 raw fish samples were purchased at the open-air fish market places in five major cities in Mazandaran Province, North of Iran. The purchased samples included *Ctenopharyngodon idella* (*n* = 135)*, Rutilus kutum* (*n* = 124), *Liza auratus* (*n =* 120), and *Hypophthalmichthys molitrix* (*n =* 109). In addition, 374 environmental and workers swab samples from workers’ hands (*n =* 96), workers’ knives (*n =* 96), work surface (*n =* 92), and containers (*n =* 90) were collected from the open-air fish markets. All of the samples were transported in ice boxes to the laboratory within 3 h after sampling. All samples were obtained with the informed consent of the workers and ethics approval for this study was granted by the Islamic Azad University, Iran.

### Isolation and detection of *Listeria* spp.

*Listeria* spp. were isolated and detected using ISO11290-1 method [22]. Briefly, samples were pre-enriched by half Fraser broth (Oxoid, Basingstoke, UK) and enriched by Fraser broth (Oxoid, Basingstoke, UK) for 48 and 24 h at 37 °C, respectively. Finally, the enriched Fraser broth-culture was streaked onto Palcam agar (Oxoid, Basingstoke, UK) and Oxford agar (Oxoid, Basingstoke, UK) followed by 24 to 48 h incubation at 37 °C. The presumed colonies were verified by biochemical tests and API *Listeria* (bioMérieux, Marcy l’Étoile, France). The isolates of *Listeria* spp. were then further confirmed by PCR [23].

### Serotyping of *L. monocytogenes* isolates

The detected *L. monocytogenes* isolates were serotyped using the commercially prepared *Listeria* antisera against somatic (O) and flagellar (H) antigens according to the manufacturer (Denka-Seiken Co. Ltd., Tokyo, Japan).

### Detection of virulence genes by multiplex-PCR

Two multiplex-PCR were used for detection of nine virulence-associated genes. Detection of *inlA, inlB, inlC,* and *inlJ* genes was performed by a multiplex-PCR using primers and cycling conditions as described by D Liu, ML Lawrence, FW Austin and AJ Ainsworth [13], and H Jamali and KL Thong [24]. The PCR assay was done as previously described for the *actA*, *hlyA*, *iap*, *plcA*, and *prfA* genes in *L. monocytogenes* isolates [25].

### Phenotypic detection of antimicrobial resistance in *Listeria* spp. isolates

Antibiotic susceptibility testing was done using the Kirby–Bauer disc diffusion method on Mueller Hinton agar (Oxoid, Basingstoke, UK) supplemented with 5 % defibrinated sheep blood [26]. Ampicillin (30 μg), chloramphenicol (30 μg), cephalothin (30 μg), cefotaxime (30 μg), ceftazidime (30 μg), cefuroxime (30 μg), erythromycin (15 μg), florfenicol (30 μg), gentamycin (10 μg), kanamycin (30 μg), pefloxacin (5 μg), penicillin G (10 unit), rifampicin (5 μg), streptomycin (30 μg), tetracycline (30 μg), trimethoprim-sulfamethoxazole (1.25/23.75 μg), and vancomycin (30 μg) were applied as antibiotic agents. As CLSI breakpoints for Listeria species only include a few antimicrobial agents such as sulfamethoxazole-trimethoprim, ampicillin, and penicillin; therefore CLSI [26] breakpoints for Enterococcus were used for the other antimicrobial agents as recommended by M Conter, D Paludi, E Zanardi, S Ghidini, A Vergara and A Ianieri [27], and Q Li, J Sherwood and C Logue [28].

### Antimicrobial resistance genes profiling of *L. monocytogenes* isolates

The tetracycline resistance genes (*tetA, tetB, tetC, tetL, tetM,* and *tetS*), ampicillin resistance gene (*ampC*), vancomycin resistance gene (*vanA* and *vanB*), erythromycin resistance gene (*ermB*), florfenicol resistance gene (*floR*), chloramphenicol resistance gene (*cmlA*), and streptomycin resistance gene (*strA,* and *strB*) were detected using PCR as previously described [29–38].

## Results and discussion

Although the people in the Northern region of Iran rely on fish as their primary animal protein source, there is still no study done on the prevalence and characterization of *L. monocytogenes* in fish and environment of fish markets. The prevalence of *Listeria* spp. in raw fish and fish markets environments in this study is presented in Table 1. Out of the 862 tested samples, 133 (15.4 %) were contaminated with *Listeria* spp., out of which 104 (78.2 %) and 29 (21.8 %) were isolated from raw fish and fish market environments, respectively. The *Listeria* spp. isolates included *L. monocytogenes* (*n* = 43, 32.3 %), *L. innocua* (*n* = 47, 35.3 %), *L. seeligeri* (*n* = 24, 18 %) and *L. ivanovii* (*n* = 19, 14.3 %). All 43 isolates of *L. monocytogenes,* identified by biochemical tests, were also confirmed using PCR.

**Table 1:** Prevalence of *Listeria* spp. in raw fish and environmental samples

	No. of samples	*Listeria* spp.	*L. monocytogenes*	*L. innocua*	*L. seeligeri*	*L. ivanovii*
*Ctenopharyngodon idella*	135	32 (23.7 %)	9 (6.7 %)	14 (10.4 %)	4 (3 %)	5 (3.7 %)
*Rutilus kutum*	124	27 (21.8 %)	14 (11.3 %)	10 (8.1 %)	1 (0.8 %)	2 (1.6 %)
*Liza auratus*	120	21 (17.5 %)	7 (5.8 %)	6 (5 %)	5 (4.2 %)	3 (2.5 %)
*Hypophthalmichthys molitrix*	109	24 (22 %)	7 (6.4 %)	4 (3.7 %)	11 (10.1 %)	2 (1.9 %)
workers’ hands	96	11 (11.5 %)	2 (2.1 %)	5 (5.2 %)	1 (1 %)	3 (3.1 %)
workers’ knives	96	3 (3.1 %)	1 (1 %)	2 (2.1 %)	0	0
Work surface	92	12 (13 %)	2 (2.2 %)	6 (6.5 %)	2 (2.2 %)	2 (2.2 %)
Containers	90	3 (3.3 %)	1 (1.1 %)	0	0	2 (2.2 %)
Total	862	133 (15.4 %)	43 (5 %)	47 (5.5 %)	24 (2.8 %)	19 (2.2 %)

 Among the 862 samples, 32 *Ctenopharyngodon idella* (23.7 %), 27 *Rutilus kutum* (21.8 %), 24 *Hypophthalmichthys molitrix* (22 %), and 21 *Liza auratus* (17.5 %) were naturally contaminated with *Listeria* spp. In addition, 12 work surface (13 %), 11 workers’ hands (11.5 %), and 3 containers (3.3 %), and 3 workers’ knives (3.1 %) harboured *Listeria* spp. Several studies on the prevalence of *Listeria* spp. in fish, fish products and environments have been performed worldwide [39, 40]. The prevalence of *Listeria* spp. isolated from raw fish and fish markets in this study concurred with the earlier findings in Iran and other countries [41, 42]. However, our findings showed higher prevalence of *Listeria* spp. compared with the previous studies [43, 44].

 In the present study, *L. monocytogenes* was isolated from 37/488 raw fish (7.6 %) and 6/374 environments of fish markets (1.6 %). Previous reports by H Momtaz and S Yadollahi [43] and VS Parihar, S Barbuddhe, M-L Danielsson-Tham and W Tham [42], indicated contamination of 7.7 and 9 % of fish samples with the pathogen*. L. monocytogenes* has been isolated from fish and fish products in earlier studies in different provinces of Iran, including Khuzestan (4 %) [41], West Azarbaijan (2.6 %) [45], and Gilan (2.2 %) [46]. Furthermore, other reports has shown contamination of fish market environment samples with the pathogen in central part of Iran (16.5 %) and Northern Greece (5 %) [40, 47]. It is known that *L. monocytogenes* is ubiquitous and can be found in soil, vegetation, sewage, surface water as well as foods [11]. Hence, it is clear that a large source of the pathogen could survive inside fish and also in its surroundings.

Of the 43 *L. monocytogenes* isolates from raw fish and environment of fish market samples, 31 (72.1 %), 10 (23.3 %), and 2 (4.7 %) were serovar 1/2a, 4b, and 1/2b, respectively (Table 2). We found that the 1/2a was the predominant *L. monocytogenes* serovar in the samples tested in the present study. The high percentage rate of serovar 1/2a in raw fish and environment of fish markets in this study is in agreement with the previous reports from Iran, Finland and Estonia [46–49]. Serovar 4b was isolated from raw fish only. The 4b was predominant serovar in fish and seafood products in Iran, China, and Israel [43, 50, 51]. On the other hand, B Siriken, ND Ayaz and I Erol [52] recently reported that serovar 1/2b was common serovar in Turkish raw and processed seafood products. Our finding on the presence of serovar 4b indicates that fish could be a potential source of human listeriosis, in the Northern region of Iran.

**Table 2:** Prevalence of *L. monocytogenes* serovars in raw fish and environmental samples

	Serovars of *L. monocytogenes*		
	1/2a	4b	1/2c
*Ctenopharyngodon idella*	6 (60 %)	3 (30 %)	0
*Rutilus kutum*	9 (64.3 %)	4 (28.6 %)	1 (7.1 %)
*Liza auratus*	6 (85.7 %)	1 (14.3 %)	0
*Hypophthalmichthys molitrix*	4 (57.1 %)	2 (28.6 %)	1 (7.1 %)
Workers’ hands	2 (100 %)	0	0
Workers’ knives	1 (100 %)	0	0
Work surface	2 (100 %)	0	0
Containers	1 (100 %)	0	0
Total	31 (72.1 %)	10 (23.3 %)	2 (4.7 %)

All 43 isolates of *L. monocytogenes* were tested for the presence/absence of virulence genes *inlA, inlB, inlC,* and *inlJ.* Although, *inlA, inlB,* and *inlC* genes were observed in all of the *L. monocytogenes, inlJ* gene was detected in 42/43 (97.7 %) of the *L. monocytogenes* isolates. The surface-associated internalin is alleged to play a role in the pathogenesis of listeriosis [53]. This is the first study, to the best of our knowledge, to examine *L. monocytogenes* isolates from raw fish and environment of fish markets for the presence of four main internalin genes (*inlA, inlB, inlC,* and *inlJ*). Similar results were obtained in the previous studies, where the tested internalin genes were present in almost all of the examined *L. monocytogenes* isolates from animals [17], human listeriosis [54], different kinds of foods [15, 18, 24] and environmental samples [55].

Although, the *actA, hlyA,* and *iap* genes were detected in all of the *L. monocytogenes* isolates, *plcA* and *prfA* genes were observed in 41 (95.3 %) and 42 (97.7 %) of the isolates, respectively. The prevalence rate of these five virulence genes in this study is in concurrence with the earlier studies in Iran [43] and India [56] where the *actA, hlyA, iap, plcA,* and *prfA* genes were observed in all the *L. monocytogenes* isolates recovered from seafood samples. The presence of nine virulence-associated genes in almost all of the *L. monocytogenes* isolates suggests that these isolates could be potentially virulent*.*

In total, 53 and 57 *Listeria* spp. isolates (39.8 and 42.9 %) were resistant to one and two antibiotics, respectively (Table 3). In addition, nine isolates of *Listeria* spp. (6.8 %) were multi-drug resistant. The most frequent antibiotic-resistance was resistance to tetracycline (23.3 %), followed by penicillin G, cephalothin (each 16.5 %), streptomycin (15.8 %), florfenicol (15 %), erythromycin (14.3 %), ampicillin (12 %), trimethoprim-sulfamethoxazole (12.5 %), ceftazidime (8.3 %), vancomycin (6 %), rifampicin (3.8 %), chloramphenicol (1.5 %), and cefuroxime (1.2 %). Out of 43 *L. monocytogenes* isolates, 15 (34.9 %), 20 (46.5 %) and 6 (6.4 %) were resistant to one, two and more than two antibiotics. The *L. monocytogenes* isolates indicated high resistance to tetracycline (27.9 %), ampicillin (20.9 %), cephalothin, penicillin G, and streptomycin (each 16.3 %). All of the *Listeria* spp. isolates were sensitive to cefotaxime, gentamicin, kanamycin, and pefloxacin.

**Table 3:** Resistance profiles of *Listeria* spp. isolated from raw fish and environmental samples

	*Listeria* spp. (*n* = 133)	*L. monocytogenes* (*n* = 43)	*L. innocua* (*n* = 47)	*L. seeligeri* (*n* = 24)	*L. ivanovii* (*n* = 19)
Ampicillin	16 (12 %)	9 (20.9 %)	5 (10.6 %)	2 (8.3 %)	0
Chloramphenicol	2 (1.5 %)	1 (2.3 %)	1 (2.1 %)	0	0
Cefotaxime	0	0	0	0	0
Ceftazidime	11 (8.3 %)	6 (14 %)	4 (8.5 %)	0	1 (5.3 %)
Cefuroxime	1 (1.2 %)	0	1 (3.6 %)	0	0
Cephalothin	22 (16.5 %)	7 (16.3 %)	11 (23.4 %)	3 (12.5 %)	1 (5.3 %)
Erythromycin	19 (14.3 %)	6 (14 %)	9 (19.1 %)	3 (12.5 %)	1 (5.3 %)
Florfenicol	20 (15 %)	6 (14 %)	11 (23.4 %)	2 (8.3 %)	1 (5.3 %)
Gentamycin	0	0	0	0	0
Kanamycin	0	0	0	0	0
Pefloxacin	0	0	0	0	0
Penicillin G	22 (16.5 %)	7 (16.3 %)	9 (19.1 %)	2 (8.3 %)	4 (21.1 %)
Rifampicin	5 (3.8 %)	1 (2.3 %)	2 (4.3 %)	2 (8.3 %)	0
Streptomycin	21 (15.8 %)	7 (16.3 %)	8 (17 %)	3 (12.5 %)	3 (15.8 %)
Tetracycline	31 (23.3 %)	12 (27.9 %)	11 (23.4 %)	5 (20.8 %)	3 (15.8 %)
Trimethoprim-sulfamethoxazole	14 (10.5 %)	5 (11.6 %)	5 (10.6 %)	3 (12.5 %)	1 (5.3 %)
Vancomycin	8 (6 %)	3 (7 %)	4 (8.5 %)	1 (4.2 %)	0
Resistant to 1 antibiotic	53 (39.8 %)	15 (34.9 %)	14 (29.8 %)	10 (41.7 %)	14 (73.7 %)
Resistant to 2 antibiotics	57 (42.9 %)	20 (46.5 %)	27 (57.4 %)	8 (33.3 %)	2 (10.5 %)
Resistant to > 2 antibiotics	9 (6.8 %)	6 (14 %)	3 (6.4 %)	0	0

The 43 *L. monocytogenes* were examined for the presence of resistance genes. Six of 43 *L. monocytogenes* isolates (14 %) harbored more than one antimicrobial resistance gene. Among the evaluated serovars of *L. monocytogenes* isolates*,* a higher prevalence of antimicrobial resistance genes was detected in serovar 1/2a (81.5 %), followed by serovar 4b (18.5 %). However, the resistance genes were not found in serovar 1/2c isolates.

For tetracycline resistance, the *tetM* and *tetA* genes were present in 91.7 and 83.3 % of the tetracycline-resistant isolates, respectively and 71.4 % of the penicillin-resistant isolates harboured *penA* gene. Out of 7 streptomycin-resistant isolates, 42.9 and 14.3 % isolates contained *strA,* and *strB,* respectively. Furthermore, the *ampC* and *vanA* resistance genes were found in 66.7 and 33.3 % of the ampicillin- and vancomycin-resistant isolates, respectively. However, the *tetB, tetC, tetL, tetS folR, cmlA,* and *vanB* were not detected in the examined *L. monocytogenes* isolates from raw fish and open-air fish market environments. The prevalence rate of the *tetM, tetA, penA* and *strA* genes in the present study is in agreement with earlier investigations, in which a high frequency of these resistance genes in *L. monocytogenes* isolates was reported by C Poyart-Salmeron, P Trieu-Cuot, C Carlier, A MacGowan, J McLauchlin and P Courvalin [57], and V Srinivasan, H Nam, L Nguyen, B Tamilselvam, S Murinda and S Oliver [29].

In the current study, the phenotypic resistance profiles of the *L. monocytogenes* isolates were not confirmed by detection of resistance genes. For instance, 2 of 6 vancomycin-resistant isolates which showed phenotypic resistance to vancomycin, harbored *vanA* gene and none of them contained *vanB.* Likewise, out of 43 *L. monocytogenes* isolates, 7 (16.3 %) were phenotypically resistant to penicillin G, however, only 6 of the isolates (11.6 %) carried *penA* resistance gene. The same results were reported in earlier studies [29]. This inconsistency suggests that mutation in ribosomal protein gene or decreased outer membrane permeability can contribute to antimicrobial resistance phenotypes [58, 59].

A high resistance of *L. monocytogenes* to tetracycline and penicillin G was observed in the present study. Our findings were in agreement with a previous investigation, in which a high resistance of *L. monocytogenes* to tetracycline and penicillin G was also reported by AA Fallah, SS Saei-Dehkordi and M Mahzounieh [47], and O Rodas-Suárez, J Flores-Pedroche, J Betancourt-Rule, EI Quiñones-Ramírez and C Vázquez-Salinas [39]. Tetracycline and fluoroquinolones are widely applied as growth supplement and therapeutic agents in Iranian fish farms, respectively. The presence of antibiotic-resistant *L. monocytogenes* as well as multi-drug resistant isolates in fish on the one hand and transmission of the pathogen through contaminated fish on the other hand, clarify major public health concerns associated with this pathogen.

## Conclusions

In conclusion, recovery of potentially pathogenic *L. monocytogenes* from raw fish and environment of open-air fish market samples evidences the zoonotic potential of listeriosis. Hence, further surveillance of the prevalence of *L. monocytogenes* and also of emerging antibiotic resistance is required to enable the recognition of the contaminated foods, as well as ensure the effective antibiotic treatment.

## References

[1] Cocolin L, Stella S, Nappi R, Bozzetta E, Cantoni C, Comi G (2005). Analysis of PCR-based methods for characterization of *Listeria monocytogenes* strains isolated from different sources. Int J Food Microbiol.

[2] Liu D, Liu D (2008). Epidemiology. Handbook of *Listeria monocytogenes*.

[3] Mead PS, Slutsker L, Dietz V, McCaig LF, Bresee JS, Shapiro C, Griffin PM, Tauxe RV (1999). Food-related illness and death in the United States. Emerg Infect Dis.

[4] FAO (1999). Fisheries report No. 604. Expert consultation on the trade impact of *Listeria* in fish products.

[5] Tham W, Ericsson H, Loncarevic S, Unnerstad H, Danielsson-Tham M-L (2000). Lessons from an outbreak of listeriosis related to vacuum-packed gravad and cold-smoked fish. Int J Food Microbiol.

[6] Kasper S, Huhulescu S, Auer B, Heller I, Karner F, Würzner R, Wagner M, Allerberger F (2009). Epidemiology of listeriosis in Austria. Wien Klin Wochenschr.

[7] Kathariou S (2002). *Listeria monocytogenes* virulence and pathogenicity, a food safety perspective. J Food Prot.

[8] Swaminathan B, Gerner-Smidt P (2007). The epidemiology of human listeriosis. Microbes Infect.

[9] Zhang Y, Yeh E, Hall G, Cripe J, Bhagwat AA, Meng J (2007). Characterization of *Listeria monocytogenes* isolated from retail foods. Int J Food Microbiol.

[10] Cartwright EJ, Jackson KA, Johnson SD, Graves LM, Mahon BE, Silk BJ (2013). Listeriosis outbreaks and associated food vehicles, United States, 1998–2008. Emerg Infect Dis.

[11] Farber J, Peterkin P (1991). *Listeria monocytogenes*, a food-borne pathogen. Microbiol Rev.

[12] Orsi R, Ripoll D, Yeung M, Nightingale K, Wiedmann M (2007). Recombination and positive selection contribute to evolution of *Listeria monocytogenes inlA*. Microbiology.

[13] Liu D, Lawrence ML, Austin FW, Ainsworth AJ (2007). A multiplex PCR for species-and virulence-specific determination of *Listeria monocytogenes*. J Microbiol Methods.

[14] Vázquez-Boland JA, Kuhn M, Berche P, Chakraborty T, Domínguez-Bernal G, Goebel W, González-Zorn B, Wehland J, Kreft J (2001). *Listeria* pathogenesis and molecular virulence determinants. Clin Microbiol Rev.

[15] Sant’Ana AS, Igarashi MC, Landgraf M, Destro MT, Franco BD (2012). Prevalence, populations and pheno-and genotypic characteristics of *Listeria monocytogenes* isolated from ready-to-eat vegetables marketed in São Paulo, Brazil. Int J Food Microbiol.

[16] Schwartz T, Kohnen W, Jansen B, Obst U (2003). Detection of antibiotic‐resistant bacteria and their resistance genes in wastewater, surface water, and drinking water biofilms. FEMS Microbiol Ecol.

[17] Jamali H, Radmehr B (2013). Frequency, virulence genes and antimicrobial resistance of *Listeria* spp. isolated from bovine clinical mastitis. Vet J.

[18] Jamali H, Radmehr B, Thong KL (2013). Prevalence, characterisation, and antimicrobial resistance of *Listeria* species and *Listeria monocytogenes* isolates from raw milk in farm bulk tanks. Food Control.

[19] Marian M, Sharifah Aminah S, Zuraini M, Son R, Maimunah M, Lee H, Wong W, Elexson N (2012). MPN-PCR detection and antimicrobial resistance of *Listeria monocytogenes* isolated from raw and ready-to-eat foods in Malaysia. Food Control.

[20] Safdar A, Armstrong D (2003). Antimicrobial activities against 84 *Listeria monocytogenes* isolates from patients with systemic listeriosis at a comprehensive cancer center (1955–1997). J Clin Microbiol.

[21] Harakeh S, Saleh I, Zouhairi O, Baydoun E, Barbour E, Alwan N (2009). Antimicrobial resistance of *Listeria monocytogenes* isolated from dairy-based food products. Sci Total Environ.

[22] ISO 11290–1 (1996). Microbiology of food and animal feeding stuffs – horizontal method for the detection and enumeration of Listeria monocytogenes – Part 1: detection method.

[23] Aznar R, Alarcón B (2003). PCR detection of *Listeria monocytogenes*: a study of multiple factors affecting sensitivity. J Appl Microbiol.

[24] Jamali H, Thong KL (2014). Genotypic characterization and antimicrobial resistance of *Listeria monocytogenes* from ready-to-eat foods. Food Control.

[25] Kalorey D, Kurkure N, Warke S, Rawool D, Malik S, Barbuddhe S (2006). Isolation of pathogenic *Listeria monocytogenes* in faeces of wild animals in captivity. Comp Immunol Microbiol Infect Dis.

[26] CLSI (2010). Methods for antimicrobial dilution and disk susceptibility testing of infrequently isolated or fastidious bacteria; approved guideline (M45-A2).

[27] Conter M, Paludi D, Zanardi E, Ghidini S, Vergara A, Ianieri A (2009). Characterization of antimicrobial resistance of foodborne *Listeria monocytogenes*. Int J Food Microbiol.

[28] Li Q, Sherwood J, Logue C (2007). Antimicrobial resistance of *Listeria* spp. recovered from processed bison. Lett Appl Microbiol.

[29] Srinivasan V, Nam H, Nguyen L, Tamilselvam B, Murinda S, Oliver S (2005). Prevalence of antimicrobial resistance genes in *Listeria monocytogenes* isolated from dairy farms. Foodbourne Pathog Dis.

[30] Gevers D, Danielsen M, Huys G, Swings J (2003). Molecular characterization of *tet(M)* genes in Lactobacillus isolates from different types of fermented dry sausage. Appl Environ Microbiol.

[31] Clermont D, Chesneau O, De Cespédès G, Horaud T (1997). New tetracycline resistance determinants coding for ribosomal protection in streptococci and nucleotide sequence of *tet (T)* isolated from *Streptococcus pyogenes* A498. Antimicrob Agents Chemother.

[32] Charpentier E, Gerbaud G, Courvalin P (1993). Characterization of a new class of tetracycline-resistance gene *tet(S)* in *Listeria monocytogenes* BM4210. Gene.

[33] Guillaume G, Verbrugge D, Chasseur‐Libotte ML, Moens W, Collard JM (2000). PCR typing of tetracycline resistance determinants (Tet A–E) in *Salmonella enterica* serotype Hadar and in the microbial community of activated sludges from hospital and urban wastewater treatment facilities in Belgium. FEMS Microbiol Ecol.

[34] Lanz R, Kuhnert P, Boerlin P (2003). Antimicrobial resistance and resistance gene determinants in clinical *Escherichia coli* from different animal species in Switzerland. Vet Microbiol.

[35] Okamoto K, Gotoh N, Nishino T (2002). Extrusion of penem antibiotics by multicomponent efflux systems MexAB-OprM, MexCD-OprJ, and MexXY-OprM of *Pseudomonas aeruginosa*. Antimicrob Agents Chemother.

[36] Sutcliffe J, Tait-Kamradt A, Wondrack L (1996). *Streptococcus pneumoniae* and *Streptococcus pyogenes* resistant to macrolides but sensitive to clindamycin: a common resistance pattern mediated by an efflux system. Antimicrob Agents Chemother.

[37] Keyes K, Hudson C, Maurer JJ, Thayer S, White DG, Lee MD (2000). Detection of florfenicol resistance genes in *Escherichia coli* isolated from sick chickens. Antimicrob Agents Chemother.

[38] Gebreyes WA, Altier C (2002). Molecular characterization of multidrug-resistant *Salmonella enterica* subsp. *enterica* serovar Typhimurium isolates from swine. J Clin Microbiol.

[39] Rodas-Suárez O, Flores-Pedroche J, Betancourt-Rule J, Quiñones-Ramírez EI, Vázquez-Salinas C (2006). Occurrence and antibiotic sensitivity of *Listeria monocytogenes* strains isolated from oysters, fish, and estuarine water. Appl Environ Microbiol.

[40] Soultos N, Abrahim A, Papageorgiou K, Steris V (2007). Incidence of *Listeria* spp. in fish and environment of fish markets in Northern Greece. Food Control.

[41] Maktabi S, Fazlara A, Ebrahimian S (2011). Incidence of *Listeria* species in farmed tropical fish in Khuzestan, Iran. World J Fish Marine Sci.

[42] Parihar VS, Barbuddhe S, Danielsson-Tham M-L, Tham W (2008). Isolation and characterization of *Listeria* species from tropical seafoods. Food Control.

[43] Momtaz H, Yadollahi S (2013). Molecular characterization of *Listeria monocytogenes* isolated from fresh seafood samples in Iran. Diagn Pathol.

[44] Rahimi E, Shakerian A, Raissy M (2012). Prevalence of *Listeria* species in fresh and frozen fish and shrimp in Iran. Ann Microbiol.

[45] Modaresi R, Mardani K, Tukmechi A, Ownagh A (2011). Prevalence of *Listeria* spp. in fish obtained from Urmia fish markets. Afr J Microbiol Res.

[46] Basti AA, Misaghi A, Salehi TZ, Kamkar A (2006). Bacterial pathogens in fresh, smoked and salted Iranian fish. Food Control.

[47] Fallah AA, Saei-Dehkordi SS, Mahzounieh M (2013). Occurrence and antibiotic resistance profiles of *Listeria monocytogenes* isolated from seafood products and market and processing environments in Iran. Food Control.

[48] Johansson T, Rantala L, Palmu L, Honkanen-Buzalski T (1999). Occurrence and typing of *Listeria monocytogenes* strains in retail vacuum-packed fish products and in a production plant. Int J Food Microbiol.

[49] Kramarenko T, Roasto M, Meremäe K, Kuningas M, Põltsama P, Elias T (2013). *Listeria monocytogenes* prevalence and serotype diversity in various foods. Food Control.

[50] Vasilev V, Japheth R, Breuer R, Andorn N, Ben Abraham R, Yoni Y, Valinsky L, Agmon V (2010). A survey of *Listeria monocytogenes* strains, isolated from ready-to-eat foods in Israel over a period of 10 years, 1998–2007. Food Control.

[51] Wang X-M, Lü X-F, Yin L, Liu H-F, Zhang W-J, Si W, Yu S-Y, Shao M-L, Liu S-G (2013). Occurrence and antimicrobial susceptibility of *Listeria monocytogenes* isolates from retail raw foods. Food Control.

[52] Siriken B, Ayaz ND, Erol I (2013). Prevalence and serotype distribution of *Listeria monocytogenes* in salted anchovy, raw anchovy, and raw mussel using IMS-based cultivation technique and PCR. J Aquat Food Product Technol.

[53] McGann P, Raengpradub S, Ivanek R, Wiedmann M, Boor KJ (2008). Differential regulation of *Listeria monocytogenes* internalin and internalin-like genes by σb and PrfA as revealed by subgenomic microarray analyses. Foodborne Pathog Dis.

[54] Mammina C, Aleo A, Romani C, Pellissier N, Nicoletti P, Pecile P, Nastasi A, Pontello MM (2009). Characterization of *Listeria monocytogenes* isolates from human listeriosis cases in Italy. J Clin Microbiol.

[55] GelbíčoVá T, KaRpíšKoVá R (2012). Outdoor environment as a source of *Listeria monocytogenes* in food chain. Czech Journal of Food Science.

[56] Das S, Lalitha KV, Thampuran N, Surendran PK (2013). Isolation and characterization of *Listeria monocytogenes* from tropical seafood of Kerala, India. Ann Microbiol.

[57] Poyart-Salmeron C, Trieu-Cuot P, Carlier C, MacGowan A, McLauchlin J, Courvalin P (1992). Genetic basis of tetracycline resistance in clinical isolates of *Listeria monocytogenes*. Antimicrob Agents Chemother.

[58] Yan W, Taylor D (1991). Characterization of erythromycin resistance in *Campylobacter jejuni* and *Campylobacter coli*. Antimicrob Agents Chemother.

[59] Farmer S, Li Z, Hancock RE (1992). Influence of outer membrane mutations on susceptibility of *Escherichia coli* to the dibasic macrolide azithromycin. J Antimicrob Chemother.

